# Characterization of neural crest-derived stem cells isolated from human bone marrow for improvement of transplanted islet function

**DOI:** 10.1080/03009734.2019.1658661

**Published:** 2019-10-18

**Authors:** Anja Brboric, Svitlana Vasylovska, Jonna Saarimäki-Vire, Daniel Espes, José Caballero-Corbalan, Gunnar Larfors, Timo Otonkoski, Joey Lau

**Affiliations:** a Department of Medical Cell Biology, Uppsala University, Uppsala, Sweden;; b Research Programs Unit, Molecular Neurology and Biomedicum Stem Cell Centre, Faculty of Medicine, University of Helsinki, Helsinki, Finland;; c Department of Medical Sciences, Uppsala University, Uppsala, Sweden

**Keywords:** Adult bone marrow, diabetes, islet transplantation, neural crest stem cells, pluripotent stem cells

## Abstract

**Background:** Murine boundary cap-derived neural crest stem cells (NCSCs) are capable of enhancing islet function by stimulating beta cell proliferation as well as increasing the neural and vascular density in the islets both *in vitro* and *in vivo*. This study aimed to isolate NCSC-like cells from human bone marrow.

**Methods:** CD271 magnetic cell separation and culture techniques were used to purify a NCSC-enriched population of human bone marrow. Analyses of the CD271+ and CD271- fractions in terms of protein expression were performed, and the capacity of the CD271+ bone marrow cells to form 3-dimensional spheres when grown under non-adherent conditions was also investigated. Moreover, the NCSC characteristics of the CD271+ cells were evaluated by their ability to migrate toward human islets as well as human islet-like cell clusters (ICC) derived from pluripotent stem cells.

**Results:** The CD271+ bone marrow population fulfilled the criterion of being multipotent stem cells, having the potential to differentiate into glial cells, neurons as well as myofibroblasts *in vitro*. They had the capacity to form 3-dimensional spheres as well as an ability to migrate toward human islets, further supporting their NCSC identity. Additionally, we demonstrated similar migration features toward stem cell-derived ICC.

**Conclusion:** The results support the NCSC identity of the CD271-enriched human bone marrow population. It remains to investigate whether the human bone marrow-derived NCSCs have the ability to improve transplantation efficacy of not only human islets but stem cell-derived ICC as well.

## Introduction

Clinical islet transplantation is an alternative treatment for a subgroup of type 1 diabetes patients suffering from severe hypoglycemic attacks and can temporarily cure the disease. However, the islet transplantation has its limitations since it requires lifelong immunosuppressive therapy, which comes with a number of complications (1). Although the results after islet transplantation are improving, the success rate is still not optimal, and insulin independence at 5 years post-transplantation is 50% at best (2–5). Several factors contribute to the loss of graft viability and function after transplantation, such as a defective engraftment process, including poor revascularization, as well as an unsuitable microenvironment at the transplantation site (6–10). Poor oxygenation of the islets and amyloid formation result in cell dysfunction and even cell death, contributing to the declining function of the graft over time (11–14).

New ways to improve islet engraftment are needed in order to make islet transplantation more successful. Several approaches, including stem cell stimulation (mesenchymal stem cells being the most extensively studied) as well as the use of alternative islet implantation sites, have been evaluated (15–17). We have previously shown that neural crest stem cells (NCSCs) from murine boundary cap have positive effects on islets after co-culture and transplantation. Moreover, co-culture and transplantation of murine NCSCs in combination with murine or human islets were shown to stimulate beta cell proliferation and increase the neural and vascular density in the islets both *in vitro* and *in vivo*. NCSCs, in contrast to mesenchymal stem cells, may potently stimulate beta cell proliferation and improve re-innervation (18–21). However, difficulties with the clinical implementation of murine NCSCs have prompted the investigation of an adult human source of NCSCs with similar characteristics.

Nagoshi et al. were the first to report the presence of NCSCs in the bone marrow of adult rodents, suggesting that a portion of the mesenchymal stem cells in the bone marrow is of neural crest lineage (22). Limited evidence exists regarding their correspondence in human bone marrow (23). Mesenchymal stem cells have been described to constitute 0.001-0.01% of bone marrow, and NCSCs are suggested to be a portion of these, consequently making it a real challenge to purify the NCSC population (24).

Low-affinity nerve growth factor receptor (CD271 or P75^NTR^) is a common receptor of neurotrophins and is required for the development and survival of peripheral neurons. It is widely expressed in the developing nervous system and is expressed by many neuronal cell types, including NCSCs (25,26). CD271 enrichment has been used for selection of both multipotent mesenchymal stem cells as well as NCSCs (27,28).

In the present study, we tested the hypothesis that human bone marrow could be used as a source of adult NCSC-derived cells. We investigated the potential of using magnetic cell separation with CD271 microbeads to label adult bone marrow NCSCs with high purity, suggesting that a positive selection would result in a more NCSC-like population of human bone marrow stromal cells. Moreover, formation of spheres has classically been used to enrich and test NCSC-like potential (29). As evidence of the NCSC-like properties of the CD271+ cell population, we assessed sphere generation and neuronal differentiation potential, both previously described as characteristic of NCSCs. Furthermore, studies suggest that the NCSC-stimulation of beta cell proliferation can only be prompted in direct contact with islets, and this through mutual migration (30,31). Since migration capacity appears to be a vital NCSC characteristic in this case, we investigated the migration behavior of the CD271+ cells, not only toward islets but toward islet-like cell clusters (ICC) as well. ICC derived from pluripotent stem cells is a promising candidate for beta cell replacement therapies (32,33). Due to the rapid progress and development of these cells, we decided to investigate the migration capacity of CD271+ cells toward ICC as well, postulating that migration tendencies toward ICC could ultimately result in positive effects.

## Materials and methods

### Ethics approval

The study was approved by the Regional Ethics Review Board, Uppsala, Sweden (2013/196; 2017/283). Informed consent was obtained from all participants included in the study.

### Human islets

Human islets were generously provided from the human islet isolation facility of the Nordic Network for Islet Transplantation (Rudbeck Laboratory, Uppsala University Hospital, Uppsala, Sweden). Islets were handpicked and cultured free-floating in CMRL 1066 medium (Thermo Fisher Scientific, Waltham, MA, USA) supplemented with L-glutamine (2 mmol/L; Sigma-Aldrich, St Louis, MO, USA), benzylpenicillin (100 U/mL; Roche Diagnostics, Scandinavia, Bromma, Sweden), and 10% fetal calf serum (Sigma-Aldrich) at 37 °C in a humidified incubator with 5% CO_2_ and 95% air.

### Human ICC derived from stem cells

Human ICC were differentiated as described previously (34), with the following modifications: cells were dislodged and dissociated during stage 4 and seeded in AggreWell™400 (STEMCELL Technologies, Vancouver, BC, Canada) to form uniform aggregates with similar size. During stage 6, the aggregates were dislodged from the AggreWells and cultured in suspension during the rest of the differentiation protocol. The time points and components were optimized during stages 6 and 7 to generate functional beta cells *in vitro* (Table 1).

**Table 1.** Differentiation protocol to generate ICC from human pluripotent stem cells (hPSCs).

Monolayer					Suspension	
ST1	ST2	ST3	ST4	ST5	ST6	ST7
(3 d)	(3 d)	(2 d)	(3 d)	(4 d)	(14 d)	(14 d)
AA CHIR	Vit C FGF7	Vit C FGF7 SANT1 RA LDN TBP EGF Nic AA	SANT1 RA LDN GC1 GSiXX ALK5i BTC	SANT1 RA LDN GC1 GSiXX ALK5i BTC	LDN GC1 GSiXX ALK5i	T3 ZM NAC

AA: activin A; ALK5i: selective inhibitor of TGFβR-1/ALK5; BTC: betacellulin; CHIR: CHIR-99021 GSK-3 inhibitor; EGF: epidermal growth factor; FGF7: fibroblast growth factor 7; GC1: high-affinity thyroid receptor α (TRα) and TRβ agonist; GSiXX: γ-secretase inhibitor; LDN: LDN-193189 Alk inhibitor; NAC: N-acetylcysteine; Nic: nicotinamide; RA: retinoid acid; SANT1: N-(4-benzylpiperazin-1-yl)-1-(3,5-dimethyl-1-phenyl-1H-pyrazol-4-yl)methanimine; ST1-7: Stage 1–7; T3: liothyronine; TBP: alpha-amyloid precursor protein modulator; Vit C: ascorbic acid; ZM: ZM 447439 Aurora inhibitor.

For morphological characterization, the ICC were fixed with 4% paraformaldehyde, sectioned, and stained for immunohistochemistry. Cryosections (7 μm) were stained with primary antibodies for insulin (polyclonal guinea pig, dilution 1:400; Fitzgerald, Acton, MA, USA), somatostatin (polyclonal rabbit, dilution 1:400; Dako North America Inc., Carpinteria, CA, USA), and glucagon (mouse biotin-conjugated, dilution 1:200; Thermo Fisher Scientific) and incubated at 4 °C overnight. Thereafter, secondary antibodies were applied and incubated for 1 h at room temperature. Secondary antibodies used were Alexa Fluor 488 anti-guinea pig (1:300; Jackson Immunoresearch Laboratory, West Grove, PA, USA), Alexa Fluor 647 donkey anti-rabbit (1:300; Jackson Immunoresearch Laboratory), and Cy™3-conjugated streptavidin (1:300; Jackson Immunoresearch Laboratory), respectively. Nuclei were stained with Hoechst (1:10,000; Thermo Fisher Scientific) and coverslips mounted with Fluorescence Mounting Medium (Dako North America Inc.).

### Preparation of NCSCs

Bone marrow aspirate was obtained from the posterior iliac crest of five healthy male volunteers aged 19–69 (median age 26 years). Mononuclear cells from bone marrow aspirate were first isolated with SepMate PBMC isolation tubes (STEMCELL Technologies) according to the manufacturer’s instructions. The supernatant was discarded and the pellet re-suspended in alpha-MEM medium (Thermo Fisher Scientific) with 10% mesenchymal stem cell fetal bovine serum (MSC FBS; Thermo Fisher Scientific) and 1% penicillin-streptomycin solution (Thermo Fisher Scientific) and seeded in 75 cm^2^ flasks at approximately 800,000 cells/flask at 37 °C in 5% CO_2_ and 95% air. Since stromal cells are known to adhere to plastic, subsequent plastic adherence technique and culture followed. Cells were allowed to adhere and propagate. The medium was changed every other day. After 10–14 days, when the cells were confluent, the cells were dispersed into single cells using TrypLE Express (1X; Thermo Fisher Scientific). CD271+ cells were separated from the single cell suspension using the CD271 MicroBead Kit according to the manufacturer’s instructions (Miltenyi Biotec, Auburn, CA, USA). The CD271+ cells were magnetically labeled with CD271 MicroBeads. The cell suspension was put into a MACS column (Miltenyi Biotec), which was placed in the magnetic field of a MACS Separator (Miltenyi Biotec). The labeled CD271+ cells were retained in the column while the negative cells passed though. The cells were manually counted before and after magnetic separation, and the fraction of CD271+ cells was calculated and is presented as mean ± SEM. The CD271+ cells were seeded in 75 cm^2^ flasks at approximately 500,000 cells/flask and cultured in DMEM/F12 medium (1:1; Thermo Fisher Scientific) supplemented with B27 (20 ng/mL; Thermo Fisher Scientific), N2 (20 ng/mL; Thermo Fisher Scientific), epidermal growth factor (EGF; 20 ng/mL; R&D Systems, Minneapolis, MN, USA), and basic fibroblast growth factor (bFGF; 20 ng/mL; R&D Systems), which has been shown to further promote the NCSC propagation (35). The medium was changed every second day until the cells grew confluent. All experiments were carried out on cells at passages 1–4.

Portions of the bone marrow cells, both before and after separation, were seeded onto coverslips in a 24-well plate to evaluate the magnetic cell separation. Coverslips were first pretreated in a 24-well plate for 10 min at 37 °C with poly-L-ornitin (Sigma-Aldrich) followed by washing with Milli-Q water and coating with laminin (20 μg/mL; Sigma-Aldrich) at 37 °C for a minimum of 4 h. A single cell suspension was prepared using TrypLE Express (Thermo Fisher Scientific), and approximately 50,000 cells were seeded into each well. After culture for approximately 1 week, the coverslips were washed with PBS (Thermo Fisher Scientific), fixed with 4% paraformaldehyde and incubated with primary antibody anti-nerve growth factor receptor (P75^NTR^) (rabbit polyclonal, dilution 1:250; Merck Millipore, Burlington, MA, USA) at 4 °C overnight. Secondary antibody Alexa Fluor 488 donkey anti-rabbit diluted 1:500 (Thermo Fisher Scientific) was added, and the coverslips with attached cells were incubated for 1 h in room temperature. Nuclei were stained with Hoechst (1:10,000; Thermo Fisher Scientific) and coverslips mounted with Fluorescence Mounting Medium (Dako North America Inc.).

### Functional characterization

For differentiation and functional characterization of the CD271+ and CD271− bone marrow cells, coverslips were pretreated and coated as described above. A single cell suspension of the cells was prepared using TrypLE Express (Thermo Fisher Scientific), and approximately 50,000 cells were seeded into each well. Thereafter, 2 mL of differentiation medium were added to the wells. Cells were cultured for at least 7 days. The differentiation medium consisted of 50% DMEM/F12, 50% neurobasal (Thermo Fisher Scientific) supplemented with N2 and B27 and was changed every other day. After culture, the coverslips were washed with PBS (Thermo Fisher Scientific), fixed with 4% paraformaldehyde, and immunostainings were performed in the same manner as described above. Primary antibodies used were anti-glial fibrillary acidic protein (GFAP) (rabbit polyclonal, dilution 1:500; Dako North America Inc.), anti-nestin (chicken polyclonal, dilution 1:1000; Bio-Techne, Minneapolis, MN, USA), SOX10 (goat polyclonal, dilution 1:50; Santa Cruz Biotechnology, Inc., Dallas, TX, USA), beta-3 tubulin (mouse polyclonal, dilution 1:1000; Thermo Fisher Scientific), and anti-alpha smooth muscle actin (SMA) (mouse monoclonal, dilution 1:400; BioLegend, San Diego, CA, USA). Then, secondary antibodies Alexa Fluor 488 donkey anti-rabbit (1:300; Jackson Immunoresearch Laboratory), Alexa Fluor 488 donkey anti-chicken (1:300; Jackson Immunoresearch Laboratory), Alexa Fluor 555 donkey anti-goat (1:500; Thermo Fisher Scientific), Alexa Fluor 488 donkey anti-mouse (1:300; Jackson Immunoresearch Laboratory), and Alexa Fluor 647 donkey anti-mouse (1:500; Thermo Fisher Scientific), respectively, were added. Nuclei were stained with Hoechst (1:10,000; Thermo Fisher Scientific) and coverslips mounted with Fluorescence Mounting Medium (Dako North America Inc.). To evaluate the differentiation capacity in the CD271+ population, the fraction of differentiated cells was counted in five non-overlapping microscopic fields for each coverslip in three separate experiments (*n* = 3). One CD271-cell fraction was evaluated as control. Values are expressed as means ± SEM.

### Confocal imaging

Images were obtained by confocal microscope Zeiss LSM 780 (Carl Zeiss Microscopy GmbH, Jena, Germany). For image analysis, Zeiss ZEN software (blue edition) was used.

### Migration assay

Since boundary cap NCSCs are known to migrate toward islets, the migration behavior of the CD271+ cells was evaluated. CD271+ cells were co-cultured with either human islets or human ICC derived from pluripotent stem cells. A drop of approximately 50,000 cells and 2–3 islets were placed on opposite sides of a coverslip placed in a 4-well plate at a distance of approximately 1000 µm. The cells were cultured in medium suitable for islet culture. Coverslips were pretreated with Laminin LN521 (BioLamina AB, Sundbyberg, Sweden) for 2 h at 37 °C in 5% CO_2_ and 95% air. To visualize the cells, they were stained with CellTracker Fluorescent Probes (1:1000; Green CMFDA; Thermo Fisher Scientific). Images were taken on days 1–7 of co-culture, and the distance between cells and islets or ICC was measured. Experiments were repeated with three biological replicates, and the migration capacity toward human islets and ICC was compared and evaluated by measuring the distance to the CD271+ cells.

### Sphere-forming capacity

The ability of the CD271+ human bone marrow cells to form free-floating 3-dimensional spheres from single cells was investigated. A single cell suspension was prepared using TrypLE Express (Thermo Fisher Scientific), and the cells were transferred to a low-adhesion 6-well plate (Corning, Corning, NY, USA) for further culture using the same culture conditions as described above.

## Results

### Morphological characterization of ICC

**Figure 1.**
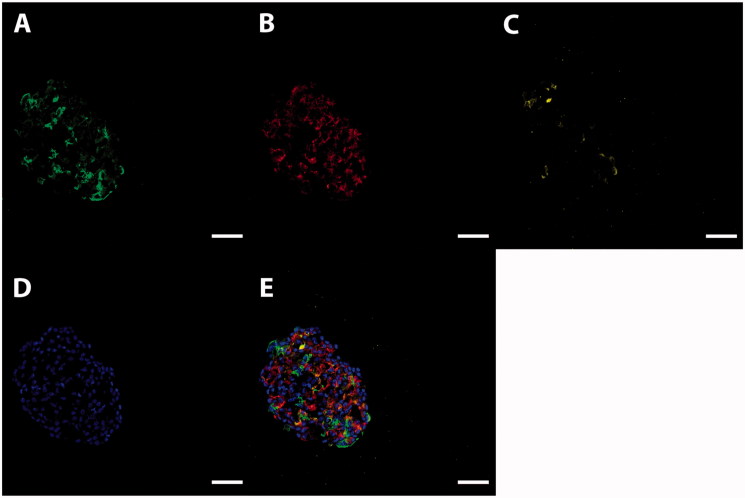
Characterization of human stem cell-derived ICC. Representative images of a sectioned islet-like cell cluster (stage 7) stained for insulin (A, green), glucagon (B, red), and somatostatin (C, yellow). Hoechst-staining is shown in blue (D). Overlay image of images A–D is shown in E. Scale bar, 50 µm.

For morphological characterization of the cells generated with the modified differentiation protocol, we performed immunostainings of ICC for pancreatic islet markers. It was found that the ICC expressed insulin, glucagon, and somatostatin (Figure 1).

### Magnetic cell separation

**Figure 2.**
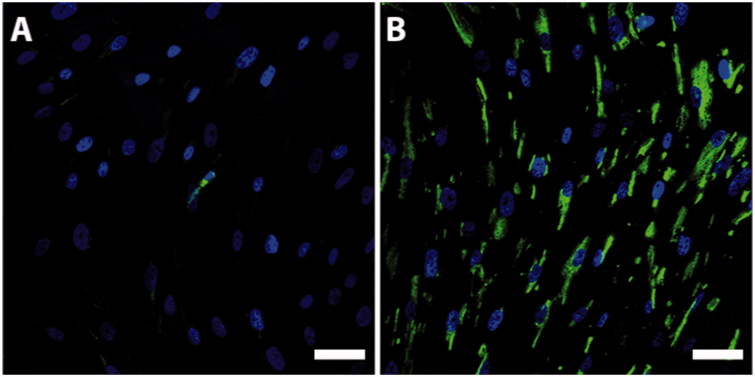
P75-staining of the bone marrow stromal cell culture before (A, green) and after (B, green) CD271 magnetic cell separation. Nuclei were counterstained with Hoechst (blue). The fraction of cells staining positive for P75 after CD271 magnetic cell separation was substantially increased. Scale bar, 50 µm.

A primary population of human bone marrow stromal cells was subsequently cultured for 2 weeks before CD271 magnetic cell separation was performed. The primary population consisted of a heterogeneous population and a small fraction of CD271+ cells (Figure 2(A)). By using the CD271 magnetic cell separation method, we were able to obtain a CD271-enriched population of bone marrow stromal cells (Figure 2(B)). The fraction of CD271+ cells in the primary population of human bone marrow stromal cells was 1.9% ± 0.6% (*n* = 5).

### Differentiation capacity

**Figure 3.**
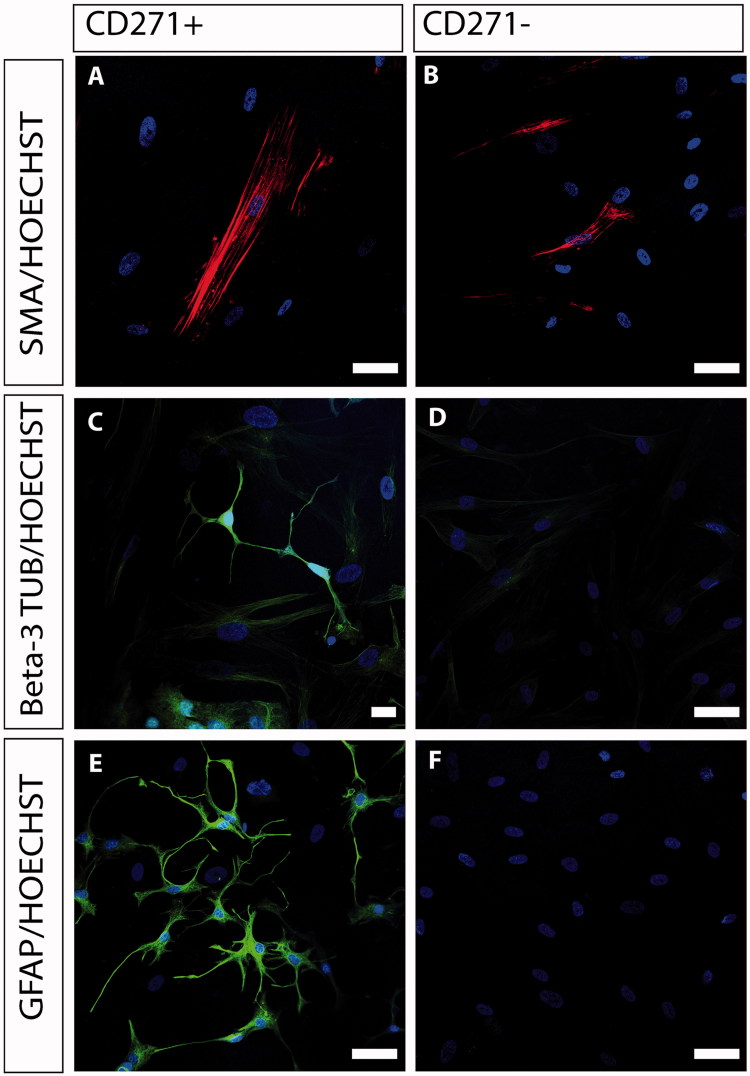
Immunocytochemical characterization of CD271+ (A, C, E) and CD271- (B, D, F) bone marrow stromal cells. CD271- cells were able to differentiate into smooth muscle cells, showing positive expression of SMA (B, red), but showed little or no expression of beta-3 tubulin and GFAP, and were thus unable to differentiate into neurons and glia cells, respectively. The CD271+ cells expressed SMA (A, red), beta-3 tubulin (C, green), and GFAP (E, green). Nuclei were counterstained with Hoechst (blue). Scale bar in A, B, D, E, and F, 50 µm. Scale bar in C, 20 µm.

**Figure 4.**
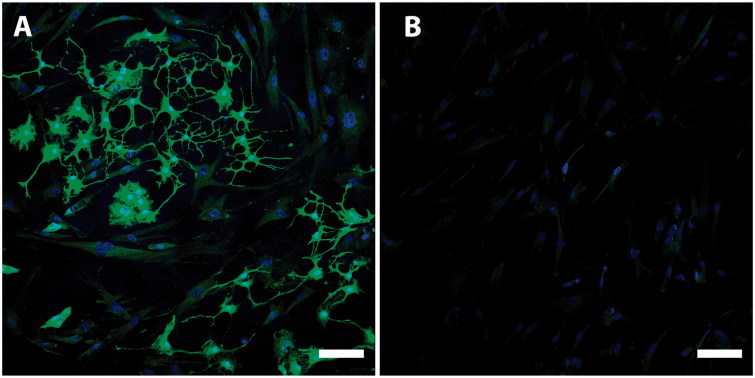
Nestin is a marker of immature and undifferentiated neuroepithelial cells. It has been suggested that nestin expression is a necessity for differentiation into neurons and glia cells. This picture demonstrates different levels of nestin expression in CD271+ (A, green) and CD271- (B, green) populations. Nuclei were counterstained with Hoechst (blue). Scale bar, 100 µm.

**Figure 5.**
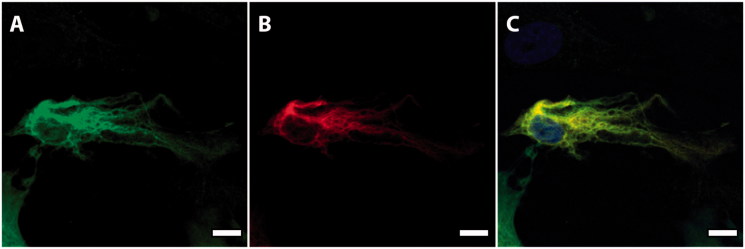
A single cell showing co-expression of nestin (A, green) and GFAP (B, red), demonstrating glial differentiation of a nestin-positive cell. C: Merged picture. Nuclei were counterstained with Hoechst (blue). Scale bar, 50 µm.

After only 1 week of culture in medium promoting differentiation, there were cells with different morphological appearances in the CD271+ cultures. There were small spindle cells, much like glia cells. Moreover, cells with long processes like neurons as well as large flattened cells consistent with myofibroblasts were observed, all with different levels of protein expression. Cells in the CD271+ population expressed both GFAP and beta-3 tubulin (Figure 3). However, there was a noteworthy difference in the differentiation capacity between donors. Quantification of the immunocytochemical staining from three different donors showed that GFAP expression was found in 24% ± 10% of the cells, and beta-3 tubulin expression was found in 25% ± 7% of cells. The CD271- cells were not able to differentiate, neither into glia cells nor neurons, showing no expression of GFAP nor beta-3 tubulin, respectively, although cultured under the same conditions (Figure 3). Both CD271+ and CD271− cell fractions were able to differentiate into myofibroblasts being positive for SMA, when cultured under neural differentiation conditions (Figure 3). In our cell cultures, nestin expression was present in most of the cells of all three donors in the CD271+ population, and the same held true for the CD271− population. However, the level of expression varied in the culture and was considerably higher in the CD271+ population (Figures 4 and 5). There was no expression of early neural crest transcription factor SOX10 in either population (data not shown).

### Migration

**Figure 6.**
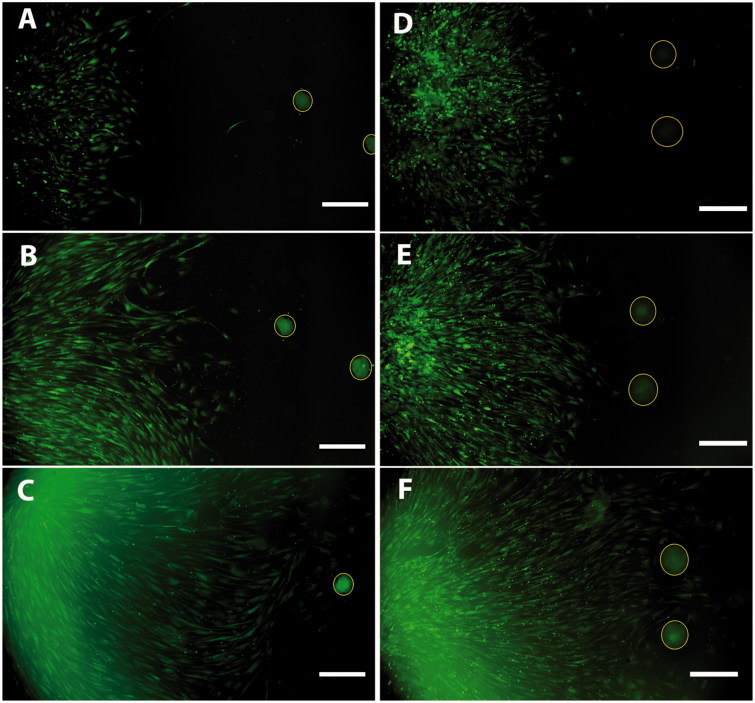
Co-culture of CD271+ cells and islets (A, B, C) or ICC (D, E, F) showed directed migration toward islets as well as ICC. Images were taken at day 1 (A, D), day 3 (B, E), and day 7 (C, F) of co-culture. CD271+ cells in green; islets and ICC encircled in yellow. Scale bar, 500 µm.

**Figure 7.**
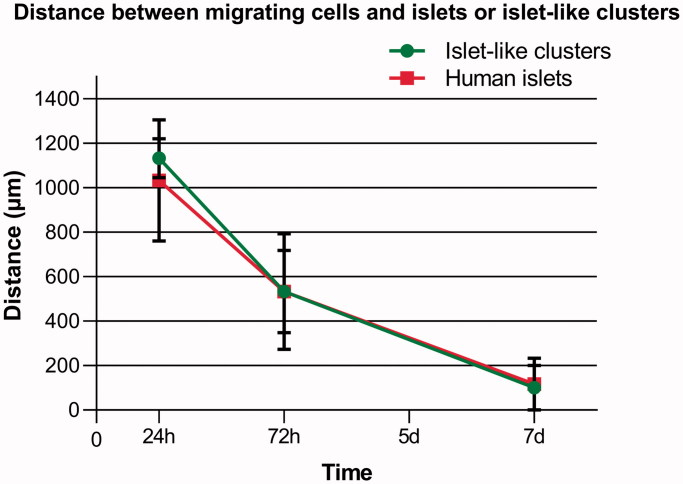
Overview of distance measured between migrating cells and islets (red) or ICC (green) in co-cultures. The CD271+ cells showed directed migration toward both islets and ICC, which is essential for stimulation of beta cell proliferation. The distance between the cells was measured at days 1, 3, and 7. The CD271+ cells were able to reach and enfold both the islets and ICC within 7 days of co-culture. Results are given as means ± SEM (*n* = 3).

The CD271+ cells migrated toward the human islets as well as the human ICC when kept in co-culture. During the following days, the distance between the cells and islets or ICC decreased. Within the first day of co-culture, the cells had attached to the laminin-coated coverslips, and by day 3 they had migrated more than half the distance toward the islets or ICC (Figure 6(B,E)). We found that the cells reached and started to enfold the islets as well as the ICC within 7 days of co-culture (Figure (C,F)). There was no obvious difference in migration behavior toward islets and ICC (Figure 7).

### Sphere formation

**Figure 8.**
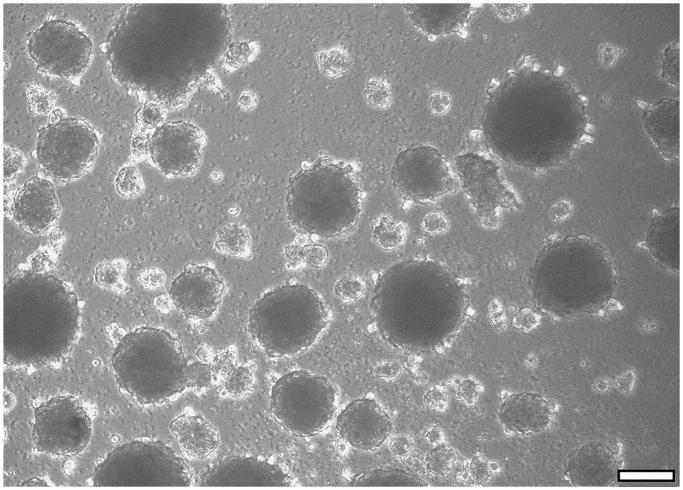
Sphere-forming capacity is seen as a classical characteristic of NCSC-like cells. CD271+ cells were able to form spheres when cultured under non-adherent conditions. After 14 days in culture, the majority of cells started to form spheres. Scale bar, 100 µm.

Within just a couple of days of culture under non-adherent conditions, 3-dimensional spheres started to form from single cells. After 2 weeks of culture, the vast majority of cells had formed spheres (Figure 8).

## Discussion

The ultimate goal of this study was to find an adult human source of cells with similar characteristics as murine boundary cap NCSCs. Such cells should have the ability to be easily harvested and expanded for the purpose of using them in clinical islet transplantation to improve the survival and function of the transplant. In the present study, we demonstrate a successful method for isolation of NCSC-like cells from human bone marrow, opening the possibility of autologous transplantation. The cells were classified as NCSC-derived based on their tri-lineage differentiation (neurons, glial cells, and myofibroblast) as well as their ability to form spheres, further proving their clonogenic potential.

Previous studies have shown the importance of the neural crest and derivatives to the pancreatic development including regulating the size of the beta cell mass and may therefore play a natural role in protecting the pancreatic islets (36). Neural crest stem cells also express angiogenic and neurogenic factors such as vascular endothelial growth factor-a (VEGF-a) and glial-derived neurotrophic factor (GDNF), which have been shown to stimulate beta cell proliferation and increase islet survival post-transplantation (19,37,38). These findings indicate that the use of NCSCs as a cellular therapy could be especially beneficial.

A limited number of studies have previously reported the presence of cells with neural crest-associated characteristics in human bone marrow (23,39). The existence of inter-donor variability of neural expression has previously been reported as well and might influence reproducibility in clinical settings (39). These studies have merely cultured the bone marrow stromal cells under mesenchymal stem cell conditions before conducting experiments. To overcome this problem and to purify a NCSC-like population of human bone marrow we explored the possibility of using CD271 antibodies to isolate a NCSC-enriched population as well as using media composition promoting the propagation of NCSCs. Indeed, we isolated a population with a higher differentiation capacity to neurons and glia cells, but the difference between donors was still noteworthy. However, the majority of the cells in the CD271+ population were nestin-positive. It has been suggested that nestin expression is a prerequisite for neural differentiation (40). Furthermore, during maturation nestin expression is replaced by cell type-specific expression such as GFAP (41). Therefore, it is possible that if we had cultured our cells under differentiation conditions for a longer time period there might have been an even higher differentiation potential and subsequently lower nestin expression.

SOX10 is a well-accepted NCSC-marker in mice, so it is noteworthy that no expression of SOX10 was observed. Several studies attempting to acquire NCSCs from adult human tissues, such as oral mucosa and gingival tissue, do not reference any SOX10 expression when characterizing the obtained NCSCs (29,42). It has also been demonstrated that NCSCs from human hair follicles express SOX10 during emigration from follicles, suggesting that post-migratory cells lose their expression (43). Thus, the absence of SOX10 is reasonable in this case.

Furthermore, we were able to demonstrate a tendency of mutual migration between CD271+ cells and islets when kept in co-culture, further demonstrating their similarities with murine boundary cap NCSCs. The mechanism by which the NCSCs exert their positive effects on beta cells is not fully understood. However, studies suggest that some of the positive effects of murine boundary cap NCSCs, for instance beta cell proliferation as well as protection against cytokine induced cell death, are exerted only in direct contact with islets (35,44). *In vitro* studies showed that beta cells and NCSCs formed cadherin junctions, and it is believed that these mediate some of the positive effects of the NCSCs. Furthermore, previous studies have shown that islets promote NCSC migration, indicating that cadherin junctions may develop as a result of mutual migration by NCSCs and islets (30,31).

To evaluate whether our NCSC-derived bone marrow cells have similar migrating features as murine boundary cap NCSCs, we investigated whether these cells had the ability to migrate toward islets *in vitro*. For instance, a previous study investigating co-culture of NCSCs from hair follicles and islets showed no mutual migration or formation of cadherin junctions and consequently no increase in beta cell proliferation, demonstrating the importance of mutual migration (30). The research on inducible pluripotent stem cell (iPS)-derived insulin-producing cells has been rapidly progressing and holds great promise for the use of autologous ICC as beta cell replacement therapy within the near future (33). We therefore investigated the migration capacity of the CD271+ cells toward ICC derived from pluripotent stem cells as well. We show that the CD271+ cells migrate just as well toward human ICC, suggesting that the NCSC-derived bone marrow cells could have beneficial effects on ICC as well. Indeed, extended studies of the effects of NCSCs on islets and ICC will be required with careful characterization of NCSCs and islets/ICC before and after co-culture as well as transplantation. This method could also be used to study further the functional maturation of ICC and improve transplantation efficiency in the future.

In conclusion, NCSCs prepared from human bone marrow could possibly enhance the results of clinical islet transplantation. More efficient methods for their isolation and expansion are, however, necessary due to their scarcity in adult tissues. Here, we demonstrated that separation of human bone marrow cells labeled with CD271 allows for the selection of cells with functional characteristics much like NCSCs with a higher degree of differentiation into multiple lineages. Further studies on the interaction between human bone marrow-derived NCSCs and pancreatic islets with the optimal goal of improving clinical islet transplantation and future beta cell replacement therapies using iPS-derived insulin producing cells are highly warranted.
